# Breast self-examination as a route to early detection in a lower-middle-income country: assessing psychosocial determinants among women in Surabaya, Indonesia

**DOI:** 10.1186/s12905-022-01748-4

**Published:** 2022-05-17

**Authors:** Triana Kesuma Dewi, Robert A. C. Ruiter, Merle Diering, Rahkman Ardi, Karlijn Massar

**Affiliations:** 1 Department of Work and Social Psychology, Faculty of Psychology and Neuroscience, Maastricht University, Maastricht, The Netherlands; 2 Department of Psychology, Faculty of Psychology, Universitas Airlangga, Jl. Airlangga 4-6, Surabaya, Indonesia; 3 Department of Health Psychology and Applied Diagnostics, University of Wuppertal, Wuppertal, Germany

**Keywords:** Breast cancer, Breast self-examination, Early detection, Good health and well-being, Social determinants of health, HBM, RAA

## Abstract

**Background:**

Breast cancer has become a public health concern in Indonesia. Regular breast self-examination (BSE) is considered an important first step for its early detection, especially in countries with limited healthcare access, as it is the case in Indonesia. This study aimed to confirm and assess the psychosocial determinants of intention to perform BSE and BSE performance.

**Methods:**

The cross-sectional study was conducted on 204 women aged 18–65 years in Surabaya, Indonesia. A 64-item survey was conducted, included variables from the Reasoned Action Approach, and the Health Belief Model, presented questions about demographics, breast cancer knowledge, and behavior related to BSE.

**Results:**

Most women (72.5%) expressed intention to perform BSE; however, only 7.8% and 2.9% performed BSE per week and per month, respectively, in the past year. Breast cancer knowledge and attitudes towards BSE were uniquely associated with BSE performance. Perceived behavioral control (PBC) and BSE attitudes were unique correlates of intention. Perceived benefits and barriers and subjective norms were significantly associated with intention and BSE behavior in bivariate analyses.

**Conclusions:**

Breast screening education should incorporate strategies for improving attitudes towards BSE, PBC, and breast cancer knowledge with perceived benefits and barriers and subjective norms as relevant targets.

## Background

Breast cancer continues to be the major type of cancer among women in Indonesia, with an incidence of 30.8% of all new cancer diagnoses among women, constituting 9.6% of all cancer-related deaths [1]. Estimates indicate that approximately 70% of patients present with an advanced stage of breast cancer, which negatively impacts treatment options and prognosis [2, 3]. Therefore, early detection remains as a cornerstone for breast cancer control in Indonesia.

Mammography is the most universally accepted method of breast cancer screening and is the golden standard [4]. However, there is limited access to it in Indonesia: Mammography has not been designated as an organized national screening program and is not covered by *Badan Penyelenggara Jaminan Sosial Kesehatan* (*BPJS Kesehatan*)—the Indonesian national health insurance, making it costly and causing only a few to have access to this screening method [5]. Additionally, some limitations of mammography are also noted, i.e., poor accuracy in women with dense breast tissue, relatively high rate of false positives, personal discomfort, and limited effectivity in women under 50 years old [6–8], while most women in Indonesia are diagnosed with breast cancer precisely at these younger ages [9]. Thus, other screening methods are more appropriate in low- and middle-income countries (LMICs) where women do not have access to more advanced screening methods such as mammography [10].

Regular breast self-examination (BSE), combined with breast awareness, is one of the strategies for achieving the early detection of breast cancer. The American Cancer Society (ACS) highlights the importance of breast awareness; that is, women should be familiar with the normal condition of their own breasts and promptly report to healthcare in the case of changes [4]. Evidence from LMICs exists that regular BSE is positively associated with the identification of breast cancer in an early stage [10], which thereby improves treatment outcomes [11–13]. As such, BSE practice followed by a prompt medical professional examination in the case of detected abnormalities may serve as a viable screening method for detecting breast cancer in an early stage, allowing improved prognosis.

A thorough understanding of factors that serve as determinants of the intention to perform BSE behavior is required to identify relevant targets for intervention to promote the early presentation of breast cancer. The study used the Reasoned Action Approach (RAA) and the Health Belief Model (HBM) as conceptual frameworks. According to RAA [14], beliefs associated with a health behavior guide the decision of whether to perform such. Behavioral beliefs form attitudes; normative beliefs shape individual perceived norms; and control beliefs structure perceived behavioral control (PBC). Additionally, the HBM, which was introduced by Rosenstock [15], explains health behavior as being determined by a person’s beliefs about a disease and available strategies for reducing the occurrence of the disease. Specifically, the model proposes that if individuals perceive that they are at risk for a disease in terms of perceived severity and vulnerability, then they will be motivated to reduce the threat by performing the recommended action provided that they expect positive health outcomes and perceive no major barriers in performing the precautionary action.

Our previous quantitative and qualitative studies [16–18] provide support for the hypothesis that the RAA and HBM components are instrumental for predicting BSE practice. Interestingly, although these models do not consider knowledge an (important) determinant of behavior but a background variable, the abovementioned studies suggested that Indonesian women lack basic knowledge about breast cancer and its symptoms, BSE, and the relationship between breast cancer and BSE performance. Therefore, the current study aims to assess women’s understanding of breast cancer and BSE.

**Fig. 1:**
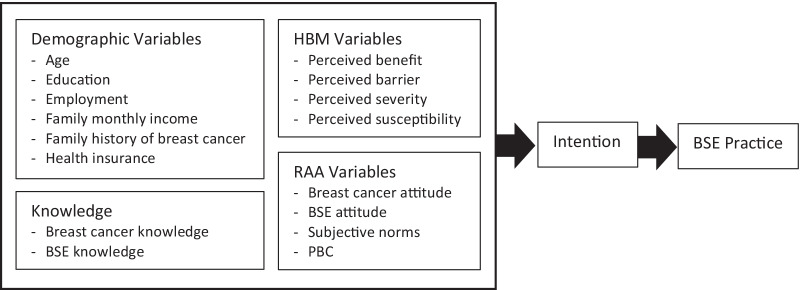
Theoretical framework for BSE practice

Specifically, the current research aims to confirm and assess the psychosocial determinants of the intention to perform BSE among Indonesian women, and the extent to which these determinants are associated with BSE performance (Fig. 1). Identifying the relevant and changeable psychological determinants of intention and BSE performance will provide insight into important targets for future educational campaigns on breast cancer awareness and stimulate regular BSE among Indonesian women [19].

## Methods

### Study design

The cross-sectional study formed a part of a larger project that aims to develop a program for the early detection of breast cancer with a focus on BSE among women in Surabaya, Indonesia. The current study was approved by the Ethical Committee of Health Research, Faculty of Public Health, Airlangga University, and the Ethics Research Committee of Psychology and Neuroscience at Maastricht University. The respondents provided written informed consent prior to participation.

### Respondents and study setting

The study was conducted in Surabaya, the capital city of East Java and the second-largest city in Indonesia, with a total population of 3,094,732 across 31 sub-districts [20]. The specific inclusion criteria included women aged (18–65 years old), who lived in Surabaya for at least one year at the time of the study, and who were never diagnosed with breast cancer. The respondents completed an online survey (constructed using Qualtrics™), which contained measures of RAA and HBM constructs related to BSE practice and questions pertaining to knowledge about breast cancer and BSE, BSE behavior, and socio-demographics. At the end of the survey, the participants were offered to join a raffle, in which they could win one of 30 packages of mobile phones/OVO/GOPAY credit worth IDR 100,000 each.

The study recruited 268 participants. After data inspection, 62 responses were considered incomplete, which were thus excluded. The final sample consisted of n = 204 respondents. Data were collected between April and May 2020.

### Materials

The questionnaire originally consisted of 72 items designed for the target variables identified in the literature and in the abovementioned qualitative studies. The RAA constructs were attitudes towards breast cancer and BSE, subjective norm and PBC towards performing BSE; the HBM constructs were composed of perceived benefit, perceived barrier, perceived severity and perceived susceptibility. Other variables included knowledge about breast cancer and BSE, BSE behavior, and socio-demographics. Table 1 lists the sample items for all constructs.

**Table 1:** Overview of psychosocial measures

Variables and sample item	Number of items	Min score	Max score	α
Intention *- I intend to perform BSE monthly as recommended*	3	1	7	.58
Attitude towards breast cancer *- I believe that BSE is related to moral wrongdoing*	3	1	7	.61
Attitude towards BSE *- I believe that BSE is easy to perform*	5	1	7	.68
Subjective norms *- I believe that most people who are important to me approve of me performing BSE monthly*	4	1	7	.85
PBC *- I am confident I can make time to perform BSE monthly*	3	1	7	.71
Perceived barriers *- I would skip BSE monthly practice if I have competing priorities, e.g., childcare, employment, family commitments*	4	1	7	.70
Perceived susceptibility *- I believe it is impossible that I would suffer from breast cancer*	3	1	7	.69
Perceived severity *- If I was diagnosed with breast cancer, I think I would have to die soon*	5	1	7	.67
Breast cancer knowledge - From what I understand, breast cancer is contagious*	23	1	23	.76
BSE Knowledge - From what I understand, BSE is painful*	7	7	49	n/a

*Reversed item

The items measuring the RAA and HBM constructs and understanding of BSE procedures were rated using a 7-point Likert-type scale ranging from 1 = strongly disagree to 7 = strongly agree. To assess the knowledge about breast cancer, the survey included 23 items pertaining to the definition, symptoms, risk factors, and screening modalities of breast cancer, which could be answered with ‘yes’, ‘no’, and ‘I don’t know’. BSE behavior was assessed using one question: ‘Did you practice BSE in the past year?’ (never; performed but not regularly; performed regularly on a monthly basis; and performed regularly on a weekly basis). Demographics recorded were age, level of education, employment, family monthly income, family history of breast cancer, and health insurance.

The questionnaire was constructed in English, translated into Bahasa and back-translated into English to ensure construct and face validity. The first and third authors were responsible for the translation process.

### Analysis

Data were tabulated in Excel worksheets and analyzed using IBM SPSS (version 23.0). After factor and reliability analyses, eight items were omitted, resulting a final set of 64 items for the main analyses. An item with communality less than 0.3 in the factor analysis and a corrected item-total correlation less than 0.3 in the reliability test was omitted.

The items reflecting the RAA and HBM constructs and breast cancer knowledge for each participant were averaged, whereas items measuring BSE knowledge were summed into a single measure to represent each psychosocial variable. Frequency distributions described the socio-demographic characteristics of the respondents. BSE performance was dichotomized into women who indicated they were BSE performers (i.e., performed BSE but not regularly; performed BSE regularly on a monthly basis; and performed BSE regularly on a weekly basis, n = 131) and those who indicated they did not perform BSE (non-performers; n = 73). Chi-square tests and independent sample t-tests were employed to compare the demographic properties and psychosocial variables, respectively. Furthermore, bivariate correlation analysis was utilized to measure the univariate association between variables. The correlations were considered to show weak, moderate, and strong associations if *r* = 0.10–0.23, *r* = 0.24–0.36 and *r* > 0.37, respectively [21]. The study used hierarchical multivariate regression analysis to assess the contribution of the variables pertaining to intention to perform BSE. Finally, hierarchical logistic regression was used to measure the unique correlates of BSE practice.

## Results

### Sample descriptives

The final sample consisted of 204 women residing in Surabaya aged 18 to 61 years (*M* = 29.72, *SD* = 8.79). The majority were employed (56.3%), achieved a higher vocational or university education (66.2%), and obtained a family monthly income equal to Surabaya’s regional standard monthly income or above (69.6%). Moreover, they had no family history of breast cancer (74%), had health insurance (72.5%), and had performed BSE in the past year (64.2%). Table 2 indicates that BSE practice is associated with age (*p* < 0.05). In other words, women in the younger age group were less likely to perform BSE compared with those in the older age group (*χ*^2^(1) = 10.12, *p* < 0.001, *V* = 0.223). However, there were no differences between BSE performers and non-performers for other demographic variables (*p* > 0.05).

**Table 2:** Socio-demographic and psychosocial characteristics of the participants

	BSE Performers		BSE Non-performers		Statistics
	n	n/N %	n	n/N %	
Age (years)					*χ*^*2*^(1) = 10.12, *p* = .001, *V* = .223*
18–30	67	32.8	54	26.5	
31–65	64	31.4	19	9.3	
Education^a^					*χ*^*2*^(1) = 2.72, *p* = .099, *V* = .116
Lower education	34	16.7	27	13.2	
Higher Education	97	47.5	46	22.5	
Employment					*χ*^*2*^(1) = 2.30, *p* = .129, *V* = .106
Unemployed (and student)	52	25.5	37	18.1	
Working	79	38.7	36	17.6	
Family monthly income^b^					*χ*^*2*^(1) = .33, *p* = .565, *V* = .04
Below regional standard	38	18.6	24	11.8	
Regional standard and above	93	45.6	49	24	
Family history of breast cancer					*χ*^*2*^(2) = .75, *p* = .684, *V* = .061
Yes	27	13.2	12	5.9	
No	96	47.1	55	27	
I don’t know	8	3.9	6	2.9	
Health insurance					*χ*^*2*^(1) = .10, *p* = .753, *V* = .022
Yes	96	47.1	52	25.5	
No	35	17.2	21	10.3	

*p < .01, *N* = 204

^a^Lower education: up to senior high school; higher education: higher vocational education or university

^b^Surabaya’s regional standard monthly income: IDR. 4,200,000

The independent sample t-tests were then conducted, which revealed that intention to perform BSE, attitudes towards breast cancer, attitudes towards BSE, perceived benefits of BSE, subjective norms towards BSE, PBC towards BSE, and breast cancer knowledge were significantly higher among BSE performers (*t*s > 2.67, *p*s < 0.01). Interestingly, the perceived barriers of performing BSE were also higher among BSE performers compared with non-performers (*t*(202) =  − 2.67, *p* = 0.008, *d* = 0.338). Table 3 provides a full overview of the results.

**Table 3:** Comparison of psychosocial and knowledge variables among performers and non-performers of BSE

	BSE performers	BSE non-performers	Statistics
	Mean (SD)	Mean (SD)	
Intention	5.63 (0.85)	5.05 (0.98)	*t*(202) = 4.45, *p* < 001, *d* = − .689*
Breast cancer attitude	6.20 (0.76)	5.81 (1.18)	*t*(202) = 2.90, *p* = .004, *d* = − .396*
BSE attitude	6.07 (0.56)	5.53 (.82)	*t*(202) = 5.63, *p* < 001, *d* = − 1.095*
Subjective norm	4.79 (1.11)	4.24 (1.17)	*t*(202) = 3.31, *p* = .001, *d* = − .423*
PBC	5.77 (.73)	5.40 (.84)	*t*(202) = 3.35, *p* = .001, *d* = − .597*
Perceived barriers	3.75 (1.12)	4.19 (1.16)	*t*(202) = − 2.67, *p* = .008, *d* = .338*
Perceived benefit	6.22 (0.57)	5.98 (.70)	*t*(202) = 2.75, *p* = .006, *d* = − .589*
Perceived susceptibility	3.93 (1.15)	3.89 (1.18)	*t*(202) = .21, *p* = .831, *d* = − .029
Perceived severity	3.93 (0.90)	4.15 (0.90)	*t*(202) = − 1.66, *p* = .099, *d* = .271
Breast cancer knowledge	15.02 (3.36)	12.34 (3.33)	*t*(202) = 5.46, *p* < 001, *d* = − .239*
BSE knowledge	32.47 (4.93)	31.31 (4.97)	*t*(202) = 1.60, *p* = .110, *d* = − .047

*﻿*p* < .01, *N* = 204

### Determinants of intentions

Bivariate correlation analysis found strong positive correlations of BSE intention to attitudes towards BSE, PBC, and perceived benefits of BSE. BSE behavior and subjective norms demonstrated a moderate positive correlation with intention, whereas perceived barriers showed a moderate negative correlation with intention. Additionally, knowledge about breast cancer and BSE indicated weak correlations with intention. Table 4 lists the full results.

**Table 4:** Correlations of intention with psychosocial variables

Psychosocial variables	M (SD)	1	2	3	4	5	6	7	8	9	10	11	12
1. Intention	5.42 (0.94)	1											
2. BSE behaviour	n/a	.299**	1										
3. Attitude towards BC	6.06 (0.94)	.086	.200**	1									
4. Attitude towards BSE	5.88 (0.72)	.475**	.369**	.315**	1								
5. Subjective norms	4.59 (1.18)	.291**	.227**	− .083	.302**	1							
6. PBC	5.64 (0.79)	.498**	.229**	.050	.477**	.489**	1						
7. Perceived barriers	3.91 (1.15)	− .294**	− .185**	− .021	− .240**	− .194**	− .324**	1					
8. Perceived benefits	6.14 (0.63)	.399**	.190**	.271**	.549**	.206**	.581**	− .265	1				
9. Perceived susceptibility	3.92 (1.16)	.016	.015	.150*	− .003	− .074	− .105	.018	− .036	1			
10. Perceived severity	4.01 (0.9)	− .112	− .116	− .179*	− .090	− .199**	− .086	.230**	− .067	.149*	1		
11. Breast cancer knowledge	14.06 (3.58)	.198**	.359**	.143*	.298**	.218**	.275**	− .203**	− .217**	.029	.038	1	
12. BSE knowledge	4.58 (0.71)	.149*	.112	.262**	.156*	− .118	.012	.018	.137	.137	.072	.111	1

*﻿*p* < .05, **p < .01, *N* = 204

Multivariate regression analysis was performed to predict intention. Age was entered into the first block, whereas psychosocial variables (breast cancer knowledge, BSE knowledge, attitudes towards BSE, perceived benefits of BSE, subjective norms towards BSE, PBC towards BSE, and perceived barriers of BSE) were entered into the second block. The full model explained 32.2% of variance in intention to perform BSE (*F*(8, 195) = 13.035, *p* < 0.001, *f*^2^ = 0.48). The study found attitudes towards BSE and PBC as unique correlates of intention; that is, respondents who displayed high levels of attitudes towards BSE (*B* = 0.34 95% *CI* = [0.14–0.53]) and PBC (*B* = 0.35, 95% *CI* = [0.16–0.55]) were more likely to indicate an intention to perform BSE (Table 5).

**Table 5:** Multiple regression analysis of demographic and psychosocial variables for predicting intention towards BSE

Variable	*B*	*B 95% CI*	*SE B*	*β*	*p*
Having older age	.09	− .15 to.33	.12	.05	.472
Breast cancer knowledge	− .01	− .04 to.03	.02	− .03	.661
BSE knowledge	.02	− .01 to.04	.01	.11	.069
Attitude towards BSE	.34	.14 to.53	.10	.26	.001**
Perceived benefit of BSE	.05	− .18 to.28	.12	.03	.682
Subjective norms towards BSE	.04	− .08 to.15	.06	.05	.524
PBC	.35	.16 to.55	.10	.30	< .001**
Perceived barriers towards BSE	− .10	− .20 to.01	.05	− .12	.056

*﻿*p* < .05, ** *p* < .01, *N* = 204, *R*^*2*^ = .322

### Determinants of BSE performance

More than one-third of the respondents (35.8%) never performed BSE in the past year. Others indicated that they performed BSE in the past year (performed but not regularly [53.4%], performed on a monthly basis [7.8%], and performed on a weekly basis [2.9%]). Bivariate correlation analysis indicated weak positive associations of BSE performance to attitudes towards breast cancer, subjective norms, PBC, and perceived benefits. Moreover, the study found a weak negative correlation between BSE performance and perceived barriers. Furthermore, the result exhibited moderate positive associations of BSE performance to breast cancer knowledge, attitudes towards BSE, and intention to perform BSE. Attitudes towards BSE and breast cancer knowledge showed strong positive correlations with BSE performance (Table 4).

Logistic regression analysis was used to elucidate the probability of BSE practice in the sample. After controlling for age (first block), the researchers added psychosocial variables with significant correlation to BSE practice into the second block. The results displayed the model significantly predicted BSE practice (*χ*^2^(9) = 56.69, *p* < 0.001) and explained 24.3%–33.3% of variance in whether participants ever utilized the screening method. The unique correlates of BSE performance were breast cancer knowledge and attitudes towards BSE (*p*s < 0.05). Respondents who indicated high levels of breast cancer knowledge (*OR* = 1.19, 95% *CI* = [1.70–1.33]) and positive attitudes towards BSE (*OR* = 2.08, 95% *CI* = [1.12–3.87]) were more likely to perform BSE. Table 6 displays the results.

**Table 6:** Logistic regression analysis of BSE practice

	*B (SE)*	*Wald*	*df*	*OR*	*OR 95% CI*	*p*
Having older age	− .33 (.39)	.75	1	.72	.34–1.52	.387
Breast cancer knowledge	.18 (.06)	10.04	1	1.19	1.70–1.33	.002**
BSE Intention	.43 (.23)	3.65	1	1.54	.99–2.40	.056
Attitude towards BC	.36 (.20)	3.16	1	1.43	.96–2.13	.076
Attitude towards BSE	.73 (.32)	5.40	1	2.08	1.12–3.87	.020*
Perceived benefit of BSE	− .34 (.37)	.83	1	.71	.35–1.48	.363
Subjective norms	.23 (.17)	1.70	1	1.26	.89–1.76	.192
Perceived behavioural control	− .10 (.32)	.10	1	.91	.49–1.68	.757
Perceived barriers towards BSE	− .11 (16)	.49	1	.89	.65–1.23	.483

Model *χ*^*2*^ (9) = 56.69, *p* < .001, *R*^2^ = .243 (Cox & Snell), *R*^2^ = .333 (Nagelkerke)

*p < .05, **p < .01, *N* = 204

## Discussion

The present study aimed to investigate the relevant and changeable psychosocial determinants that contribute to the intention and past year BSE behavior. The study included variables from the RAA [14] and HBM [15] as well as variables informed by findings of previous qualitative studies [16, 18]. Knowledge gained from the current study can inform interventions that aim to promote regular BSE practice by targeting the main explanatory factors of the (non-) performance of this preventative behavior. The results indicate that regular BSE on a monthly basis in the past year was not prevalent among the respondents, which is consistent with the previous study [17]. Additionally, the likelihood of performing BSE behavior in the past year was associated with adequate knowledge about breast cancer and positive attitudes towards BSE. Together, the two variables explained approximately 33.3% of the variance in BSE behavior.

In line with previous findings [16, 18], the current data demonstrated that breast cancer knowledge is a correlate of BSE behavior. Thus, the higher the respondents’ understanding of the nature of breast cancer in terms of definition, symptoms, risk factors, and screening modalities, the higher the likelihood they performed BSE in the past year. In the same manner, previous studies on women populations in Iran, Ghana, and Nigeria confirmed that adequate knowledge of breast cancer significantly improves the likelihood of women to perform BSE [22–24]. Together, the findings that (a) knowledge about breast cancer serves as a unique predictor of BSE performance and (b) the low rates of regular BSE practice in the current sample, imply that low BSE performance may be due to the lack of breast cancer knowledge. This conclusion is in line with that of Didarloo et al. [22], who conducted a study among students in Iran and found that respondents with high levels of knowledge about breast cancer performed BSE 5.51 times more than those with low levels of knowledge. This finding highlights the necessity of improving breast cancer literacy among Indonesian women and the need to support education on breast cancer screening.

As noted by Bartholomew Eldredge et al. [25], knowledge does not directly lead to a behavior change; women’s understanding of breast cancer alone is insufficient for the management of a BSE behavior. Ajzen et al. [26] suggested that a more positive attitude towards a health behavior play a major role in facilitating such a behavior. In line with these suggestions, the current study finds that women with positive attitudes towards BSE were 2.08 times more likely to perform the behavior than women with negative attitudes. This finding supports previous research among Indian–Australian women, which reported that respondents with positive attitudes towards general check-ups regularly practiced breast screening as recommended [27]. Conversely, women with a negative perception of BSE felt embarrassed to perform it or experienced difficulty in performing were less likely to perform BSE. This result is in line with that of Al-Dubai et al., [28], who proposed that a negative attitude towards BSE will impede BSE behavior.

The intention to perform BSE regularly among respondents was relatively high. However, only 7.8% and 2.9% of the respondents indicated that they performed BSE regularly (monthly and weekly, respectively). Additionally, although the intention was positively correlated with BSE behavior, the measure of intention failed to explain any unique variance in BSE behavior. This finding supported *intention–behavior gap hypothesis*, i.e., participants with positive intentions failed to perform the behavior [29]. Moreover, the current data indicated that the 32.2% of variance in intention to perform BSE was predicted by the positive attitudes towards BSE and a high PBC. Thus, the study concluded that women who perceived BSE as an important process and were able to perform it were more likely to report increased intention to perform such. This finding aligns with those of Wang et al. [30], who conducted a study in China, and concluded that behavioral attitude was one of the unique correlates of intention to perform breast cancer screening. Additionally, the current finding that women who felt confident with their autonomy and capacity to perform BSE indicated high levels of intention to perform such is in line with that of Roncancio et al. [31] on Latinas. The authors documented that PBC was a strong predictor of the intention to be screened for cervical cancer.

Several changeable psychosocial variables, i.e., perceived benefits, perceived barriers and subjective norms failed to explain the unique variances of intention and BSE behavior. However, in the bivariate correlation analysis, these variables were significantly associated with intention to perform BSE and BSE behavior. Similarly, previous studies reported evidence that if women perceived BSE as highly beneficial, they would be more likely to form positive intentions and/or perform BSE [17, 22, 30]. Vice versa, intention and/or BSE behavior would be less likely to occur when women perceived certain obstacles in BSE performance [17, 32, 33]. Additionally, a strong family support system characterized the Indonesian population [34]. Thus, the notion that women’s breast cancer screening behavior would be influenced by the support they (perceive to) receive from their close social circles is not surprising. Indeed, a previous study highlighted the significance of subjective norms, such as encouragement from daughters or relatives, to participate in breast screening [35]. Moreover, Cho and Lee [36] found that, in general, individuals from collectivistic cultures (i.e., Indonesia) score higher on subjective norms compare to those of individualistic culture. Therefore, the study suggests that developers of programs or interventions should focus on the role of subjective norms in the transmission of information about the advantages of performing BSE. Moreover, developers should formulate strategies for overcoming barriers to BSE performance during education on breast cancer awareness, e.g., involving women’s close friends or family in health education activities.

The study was interested in exploring the distribution of demographic and psychosocial determinants among women who performed BSE in the past year (compared with non-performers). The results indicated that women who performed BSE in the past year reported higher levels of intention to perform BSE, positive attitudes towards BSE and breast cancer (i.e., they feel that breast cancer is not related to moral wrongdoing or a taboo topic), subjective norms, and PBC compared with those of non-performers. Furthermore, BSE performers were found to possess a better understanding of breast cancer and were more likely to perceive that performing BSE is beneficial for them. Interestingly, the study also found that women who performed BSE viewed more obstacles to BSE performance compared with those who did not perform BSE. The study inferred that BSE performers gained a more realistic view of BSE performance but found it important, nonetheless, which contributed to their decision to perform. Moreover, in line with previous research, younger women were less likely to perform BSE compared with older women, perhaps because they perceived lower risk of or susceptibility to breast cancer [27].

## Conclusions

The study assessed the relevant psychosocial determinants associated with the intention to perform BSE and past performance of BSE among women in Surabaya, Indonesia. Furthermore, it confirmed the findings from the previous qualitative studies as well as the relevance of variables included in the influential models used to explain health behavior. The study provided strong evidence that improving literacy in breast cancer, including information about breast cancer symptoms and risk factors and the advantages and necessity of various forms of breast cancer screening, may serve as a basic foundation to support education interventions for breast cancer screening in Indonesia. Specifically, the study suggested that interventions should include methods and applications to strengthen women’s positive attitudes towards BSE to improve the intention to perform BSE and the likelihood of performing such a behavior. Moreover, such interventions should incorporate messages that enable the participants to generate information about effective strategies for coping with barriers that may impede BSE behavior. Finally, the study suggested that interventions should focus on increasing the knowledge and enhancing the attitudes of the social networks of women (i.e., close friends and family) because subjective norms play a significant role in the development of BSE behavior.

## Limitations

The present study has several limitations. Firstly, data were collected through a cross-sectional study, and the sample size was relatively limited. Thus, the causal relationship of the psychosocial variables to intention to perform BSE and BSE behavior could not be established. Therefore, longitudinal research that focuses on actual performed behavior should be conducted to establish such relationships. Secondly, data were collected through a self-report questionnaire. Thus, issues related to the subjectivity of a self-report measurement (e.g., social desirability bias) should also be considered in interpreting the results. Thirdly, the study sample generally achieved high levels of education, obtained considerable family incomes, was without a family history of breast cancer, and secured health insurance. Therefore, populations from different socio-economic backgrounds, such as women from lower socio-economic groups, may display different patterns of associations. Additionally, the current sample, which is highly educated, exhibited low levels of breast cancer awareness, which suggested that this deficiency may be even greater among groups from lower socio-economic brackets. Finally, the study employed an online survey and offered a lottery of financial incentives. Thus, multiple responses from a respondent might possibly exist, which potentially leads to a bias.

## Data Availability

The questionnaire and the datasets generated and/or analysed during the current study are available from https://osf.io/n5kte/.

## Abbreviations

ACS: American Cancer Society
BPJS Kesehatan: *Badan Penyelenggara Jaminan Sosial Kesehatan* (The Indonesian national health insurance)
BSE: Breast Self-examination
HBM: Health Belief Model
LMICs: Low- and Middle-Income Countries
PBC: Perceived Behavioral Control
RAA: Reasoned Action Approach
